# Analytical model for the photocurrent-voltage characteristics of bilayer MEH-PPV/TiO_2 _photovoltaic devices

**DOI:** 10.1186/1556-276X-6-350

**Published:** 2011-04-19

**Authors:** Chong Chen, Fan Wu, Hongwei Geng, Wei Shen, Mingtai Wang

**Affiliations:** 1 Institute of Plasma Physics, Chinese Academy of Sciences, Hefei 230031, PR China; 2 School of Materials Science and Engineering, Anhui University of Architecture, Hefei 230022, PR China

## Abstract

The photocurrent in bilayer polymer photovoltaic cells is dominated by the exciton dissociation efficiency at donor/acceptor interface. An analytical model is developed for the photocurrent-voltage characteristics of the bilayer polymer/TiO_2 _photovoltaic cells. The model gives an analytical expression for the exciton dissociation efficiency at the interface, and explains the dependence of the photocurrent of the devices on the internal electric field, the polymer and TiO_2 _layer thicknesses. Bilayer polymer/TiO_2 _cells consisting of poly[2-methoxy-5-(2-ethylhexyloxy)-1,4-phenylenevinylene] (MEH-PPV) and TiO_2_, with different thicknesses of the polymer and TiO_2 _films, were prepared for experimental purposes. The experimental results for the prepared bilayer MEH-PPV/TiO_2 _cells under different conditions are satisfactorily fitted to the model. Results show that increasing TiO_2 _or the polymer layer in thickness will reduce the exciton dissociation efficiency in the device and further the photocurrent. It is found that the photocurrent is determined by the competition between the exciton dissociation and charge recombination at the donor/acceptor interface, and the increase in photocurrent under a higher incident light intensity is due to the increased exciton density rather than the increase in the exciton dissociation efficiency.

## Introduction

The polymer-based photovoltaic (PV) cells consisting of conjugated polymer as electron donor (D) and nanocrystals as electron acceptor (A) are of great interest due to their advantages over conventional Si-based cells, such as low cost, easy-processability, and capability to make flexible devices [1-3]. Generally, the p-type conducting polymer acts as both electron donor and hole conductor in the photovoltaic process of the device, while the n-type semiconductor serves as both electron acceptor and electron conductor. The electron donor and acceptor can be intermixed into bulk architecture or cast into a bilayer structure in the PV devices [4-13]. The latter architecture is attractive for efficient devices, because the photogenerated electrons and holes are, to a great extent, confined to acceptor and donor sides of the D/A interface, respectively, where the spatial separation of electrons and holes will minimize the interfacial charge recombination and facilitate the transport of charge carriers toward correct electrodes with greatly reduced energy loss at wrong electrodes [1-3].

The primary processes involved in the photocurrent generation in a polymer-based PV cells include the exciton generation in the polymer after absorption of light, exciton diffusion toward the D/A interface, exciton dissociation at the D/A interface via an ultrafast electron transfer. The kinetics of the charge-carrier separation and recombination at the D/A interface imposes a great effect on the cell efficiency, and modeling the kinetics of the interfacial charge separation and recombination will offer a good way to understand the efficiency-limiting factors in the devices and to inform experimental activities. For this purpose, several theoretical models dealing with the interfacial charge separation and recombination have been developed in the past years. However, most of them are based on either Monte Carlo (MC) simulation [14-21] or numerical calculations [22,23], and only a few models offer analytical expressions [5,24-26]. Furthermore, the previous studies mainly focused on understanding the influences of interfacial dipoles [14,20], energetic disorder [15,20], light intensity [17], interface morphologies [18-22], and electrostatic interactions [20], on the interfacial charge separation and recombination at the organic/organic interfaces. The quantitative analysis of the charge transfer mechanism at the organic/inorganic interfaces in the polymer-based PV cells has been scarcely explored so far. Commonly, the photoinduced interfacial charge transfer from the polymers to inorganic semiconductors is explained by the exciton dissociation at the D/A interface due to the favorable energy match between the D and A components, without considering the role of the interfacial electric field [16,27-31]. Breeze et al [5] proposed an analytical expression including the interfacial electric field for the exciton dissociation efficiency in bilayer MEH-PPV/TiO_2 _photovoltaic device, which only expresses the dependence of exciton dissociation efficiency on the polymer layer thickness, not on the TiO_2 _layer thickness. To understand the influence of TiO_2 _layer thickness on the exciton dissociation efficiency, one needs to consider the electrical properties of the system. In other words, more factors, such as voltage drop across the TiO_2 _layer, field-dependent mobility, field-dependent exciton dissociation, and charge recombination at the D/A interface, are necessarily to be incorporated into the model.

In this article, we propose a simple analytical model to describe the exciton dissociation and charge recombination rates at the D/A interface for the bilayer MEH-PPV/TiO_2 _cells by modeling the photocurrent-voltage characteristics of the devices. Not only this model is successful in describing the effect of the internal electric field at the D/A interface on exciton dissociation efficiency, but also describes the dependence of the exciton dissociation efficiency on the polymer and TiO_2 _layer thicknesses. We verify our model by fitting the measured experimental data on bilayer MEH-PPV/TiO_2 _devices under different conditions. The results obtained from the model show that the photocurrent of the devices is determined by the competition between the exciton dissociation and the charge recombination at the D/A interface; the exciton dissociation efficiency increases with either the increase in the forward electric field or the decrease in the thicknesses of polymer and/or TiO_2 _layers. In addition, it is found that a higher incident light intensity leads to a higher photocurrent density, but a lower exciton dissociation efficiency.

## Experimental section

**Figure 1:**
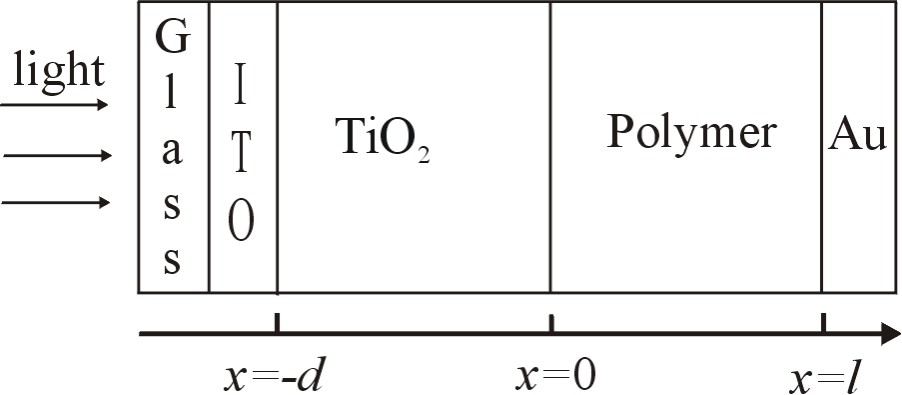
**Geometry of the bilayer device under illumination**.

**Figure 2:**
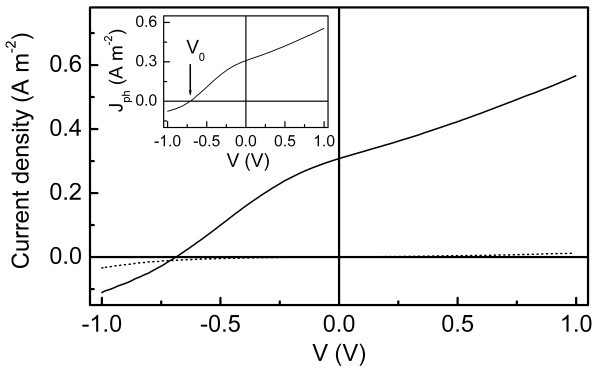
**Current-voltage characteristics of ITO/TiO**_**2**_**/MEH-PPV/Au device**. The solid line (*J*_D_) was recorded in the dark, and the dot line (*J*_L_) was measured under illumination at 470 nm with an intensity of 158.5 W/m^2^. The thickness of TiO_2 _layer was *d *= 65 nm, while that of the polymer layer was *l *= 220 nm. The inset shows the *J*_ph _as a function of bias, where the arrow indicates the compensation voltage (*V*_0_).

Poly[2-methoxy-5-(2-ethylhexyloxy)-1,4-phenylenevinylene] (MEH-PPV) (Avg. *M*_n _= 40000-70000) was purchased from Aldrich (product of USA). Titanium tetraisopropoxide [Ti(O^*i-*^Pr)_4_] (Acros, 98+%) was used as TiO_2 _precursor. The bilayer PV devices with a structure of ITO/TiO_2_/MEH-PPV/Au, as shown in Figure 1, were constructed by spinning down first a nanostructured titanium dioxide (TiO_2_) layer and then a MEH-PPV layer over indium tin oxide (ITO, ≤15 Ω/∀, Wuhu Token Sci. Co., Ltd., Wuhu, China) sheet glass, as described elsewhere [11]. The current-voltage (*J*-*V*) characteristics were measured on a controlled intensity modulated photo spectroscopy (CIMPS) (Zahner Co., Kronach, Germany) in ambient conditions. The devices were illuminated through ITO glass side by a blue light-emitting diode (LED) as light source (BLL01, *λ*_max _= 470 nm, spectral half-width = 25 nm, Zahner Co., Kronach, Germany). A reverse voltage sweep from 1 to -1 V was applied and the current density under illumination (*J*_L_) was recorded at 300 K. In order to determine the photocurrent, the current density in the dark (*J*_D_) was also recorded, and the experimental photocurrent is given by *J*_ph _= *J*_L _- J_D _[24,26,32], as shown in Figure 2. From the resulting *J*_ph_-*V *characteristics the compensation voltage (*V*_0_) was determined as the bias voltage where *J*_ph _= 0 (inset to Figure 2). During all measurements, the gold and ITO contacts were taken as negative and positive electrodes, respectively, and the effective illumination area of the cells was 0.16 cm^2^.

### The model

Since the injected charge by the electrodes can be ignored and the charge density in the bulk is low when a small voltage is applied to the device, the electric fields in the polymer (*E*_p_) and TiO_2 _(*E*_n_) regions are regarded to be constant [33]. For the small applied voltage, the internal bias in the cell is *V *- *V*_0 _[34]. Therefore, the voltage drop across the device is simply given as *E*_p_*l *+ *E*_n_*d *= *V *- *V*_0_. From the discontinuity of the electric field at the polymer/TiO_2 _interface, we have *E*_p_*ε*_p _- *E*_n_*ε*_n _= *Q *[33]. Thus, we obtain(1)(2)

where *E*_p _(*E*_n_) is the electric field in the polymer (TiO_2 _) layer, *ε*_p _(*ε*_n_) is the polymer (TiO_2_) dielectric constant, *l *(*d*) is the polymer (TiO_2_) layer thickness, and *Q *is accumulated charge density at the polymer/TiO_2 _interface.

The excitons at the D/A interface may be quenched by two processes, namely, exciton dissociation into free charge carriers and the lost of energy by luminescence or due to other processes [35-37]. Here, we only consider the exciton quenching by dissociation. Therefore, the photocurrent can be described as [38](3)

where *I *is the incident photon flux, *e *the charge of an electron, and *η*_EQE_(*V*) the voltage dependent the quantum efficiency. *η*_EQE_(*V*) can be described as [18](4)

where *η*_A _is the efficiency of photon absorption leading to the exciton generation, *η*_ED _the efficiency of excitons that diffuse to the D/A interface, *η*_CT _the efficiency of exciton dissociation by charge transfer at the D/A interface, and *η*_CC _the efficiency of charge collection at electrodes. Here, we suppose that *η*_ED _is constant, and *η*_CC _= 1 since the recombination of charges in a D/A bilayer device mainly occurs at the D/A interface [39]. In addition, we neglect the fraction of incident light reflected by the sample, then *η*_A _is taken as [40](5)

where *α *is the polymer absorption coefficient, and *L*_p _the exciton diffusion length.

**Figure 3:**
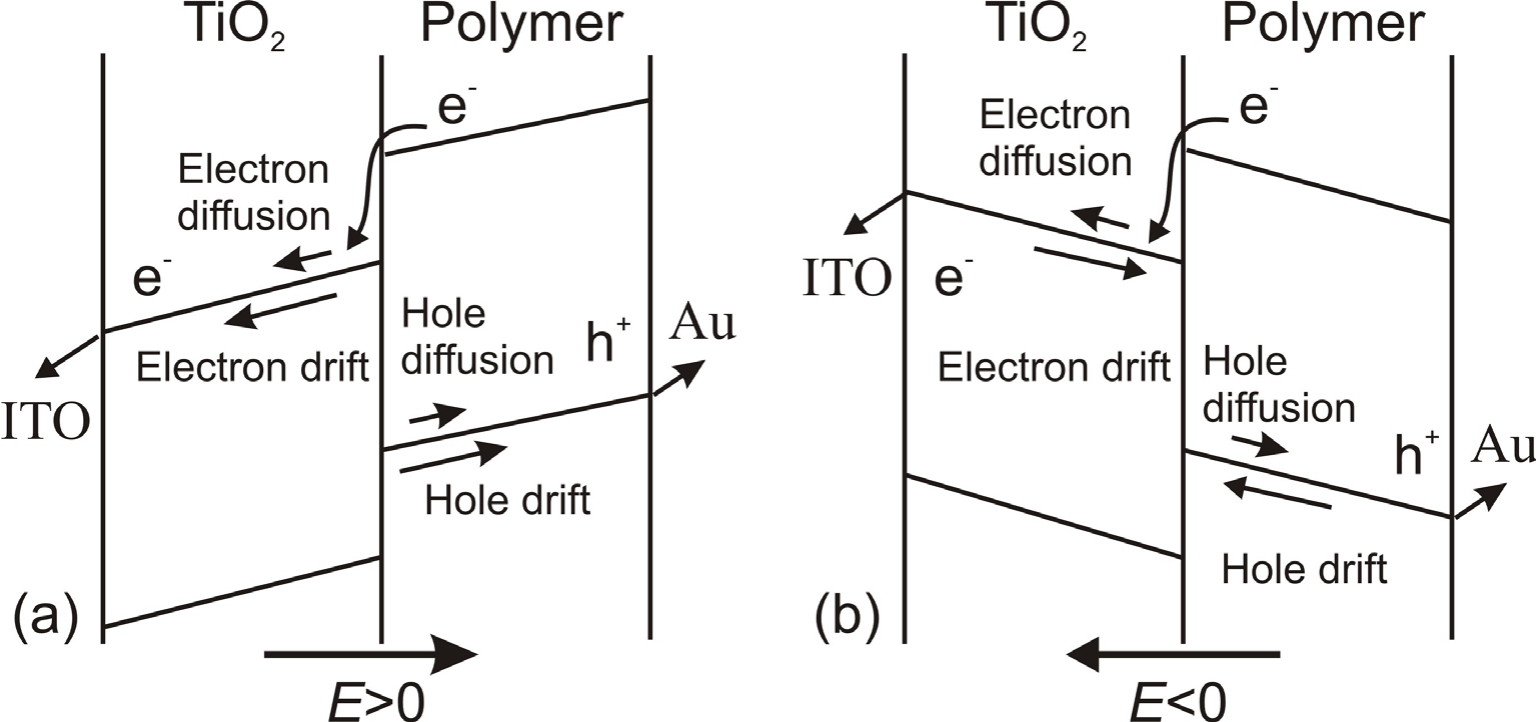
**Schematic band diagram for a bilayer TiO_2_/MEH-PPV device under (a) *E *> 0 and (b) *E *< 0**.

In a bilayer device, the electrons are injected into the acceptor layer and the holes remain in the donor layer after the interfacial exciton dissociation [39]. In other words, each charge carrier is in its respective phase. Therefore, in our case, the charge recombination in single polymer or TiO_2 _layer can be ignored. However, the recombination at the D/A interface must be considered. The presence of the internal electric field in the device may affect the charge-transport properties and also the charge recombination and exciton dissociation rates at the D/A interface. In our model, the exciton dissociation efficiency *η*_CT _is expressed in terms of the ratio between exciton recombination and separation. As shown in Figure 3a, when applying a forward internal electric field (*E *> 0), the drift and diffusion currents of the electrons (holes) in the TiO_2 _(polymer) layer are in the same direction, the electric field contributes to suppress the recombination of injected electrons in TiO_2 _with holes in the highest-occupied molecular orbital (HOMO) of the polymer by accelerating their separation at the polymer/TiO_2 _interface.

However, when applying a reverse internal electric field (*E *< 0) (Figure 3b), the drift current of the electrons (holes) in the TiO_2 _(polymer) layer is in a reverse direction, and the electric field prevents the photogenerated electrons (holes) from leaving the polymer/TiO_2 _interface, which raises the recombination of generated charge carries, i.e., reduces their separation probability at the interface. The exciton dissociation probability has a weaker dependence on the larger carrier mobility in bilayer photovoltaic devices [41]. In our case, the mobility of the electrons in the TiO_2 _layer is larger than that of the holes in the MEH-PPV layer. Therefore, the effect of the electron mobility in the TiO_2 _layer on the exciton dissociation probability is not considered in our model. Here, we define a forward hopping rate *k*_f _(*E*_p_) and a backward hoping rate *k*_b _(*E*_p_) for the holes, and the net hole hopping rate, *k*(*E*_p_), is given by their difference [42],(6)

It is known that the electric-field-dependent hole mobility has the Poole-Frenkel form [43],(7)

Here, *μ*_0 _is the zero-field mobility of holes, *γ *the electric-field-dependent parameter [44] with a value of 5 × 10^-3 ^(cm/V)^1/2 ^[45]. Assuming that the zero-field hopping rate of holes, *k*_0_, in the polymer layer is proportional to the zero-field mobility *μ*_0_, then, we get the electric-field-dependent hole hopping rate *k*(*E*) with the same form,(8)

In order to reflect the effect of an external electric field on hole transport in the polymer layer, we employ an activation energy [42]. Then, *k*_f _(*E*_p_) and *k*_b _(*E*_p_) can be expressed as, respectively(9)(10)

where *l*_0 _is the nearest neighbor hopping distance, *k*_B _the Boltzmann constant, *T *the absolute temperature, *q *the elementary charge, and *E*_a _the thermal activation energy at zero field per molecule. In our calculations, we take *E*_a _= 0.18 eV for MEH-PPV, which is comparable to the value of thermal activation energy 0.2 eV [45], and take *l*_0 _= 0.3 nm in the MEH-PPV molecules by referring to the typical distance of 0.6-1 nm between hopping sites in organic materials [46].

As *E*_p _> 0 with *E *> 0 (i.e., *V *>*V*_0_), the net hole hopping rate is equal to the excitons separation rate at the D/A interface. The exciton separation rate *k*_s_(*E*) can be derived from Equation 6-9,(11)

As mentioned above (Figure 3a), the forward electric field suppresses the recombination of the injected electrons in TiO_2 _with the holes in the polymer at the D/A interface. When the electrons transfer from TiO_2 _to the polymer layer, they have to overcome an energy barrier Δ*ϕ *at the D/A interface, in which the energy barrier is inevitably influenced by several factors, such as the applied bias, the electron-hole Coulomb interactions, and the temperature. Thus, the electron-hole recombination rate *k*_r_(*E*) (i.e., the electrons transfer rate from TiO_2 _to the polymer layer) at the D/A interface should be of an exponential dependence on the energy barrier. In addition, the recombination rate at the D/A interface should increase with temperature due to a thermally activated interfacial charge-transfer process [47]. Here, the bimolecular recombination of mobile charges and the space charge effect at the D/A interface are not considered for simplification. Furthermore, due to the large dielectric constant of TiO_2 _[47], the electron-hole Coulomb interactions can be ignored. Therefore, the energy barrier Δ*ϕ *should be dependent on the temperature *T *and the applied bias *V*. With the above considerations, we assumed a simple form for *k*_r_(*E*) [45],(12)

When *V *= 0 V, *k*_r_(*E*) = *v*_0_. Thus, *v*_0 _is a zero-field recombination rate constant that depends on the used materials and the thickness of the polymer (TiO_2_) film in the devices, and the energy barrier Δ*ϕ *is the potential energy determined by the applied bias *V*. In order to get *k*_r_, it is assumed that Δ*ϕ *is in direct proportion to *V*^*λ*^, i.e., Δ*ϕ *= *βV*^*λ*^*q*, where *β *is a proportionality factor and *λ *is used to characterize the bias-dependent strength of Δ*ϕ*. Here, it should be noted that Δ*ϕ *in a specific device may not be in proportional to *V *(i.e., *λ *≠ 1) because the bias-dependent strength of should be determined by experimental results. Moreover, Δ*ϕ *has the dimensions of energy, thus *β *is not a dimensionless factor. Finally, according to Equation 12 and the expression of *k*_r _can be expressed as,(13)

Equation 13 shows that *k*_r_(*E*) decreases with increasing the forward applied bias. Hence, the exciton dissociation efficiency *η*_CT _is [24,26,48],(14)

The photocurrent *J*_ph _for *V > V_0 _*can be derived from Equations 3-5 and 14 as follows:(15)

## Results and discussion

**Figure 4:**
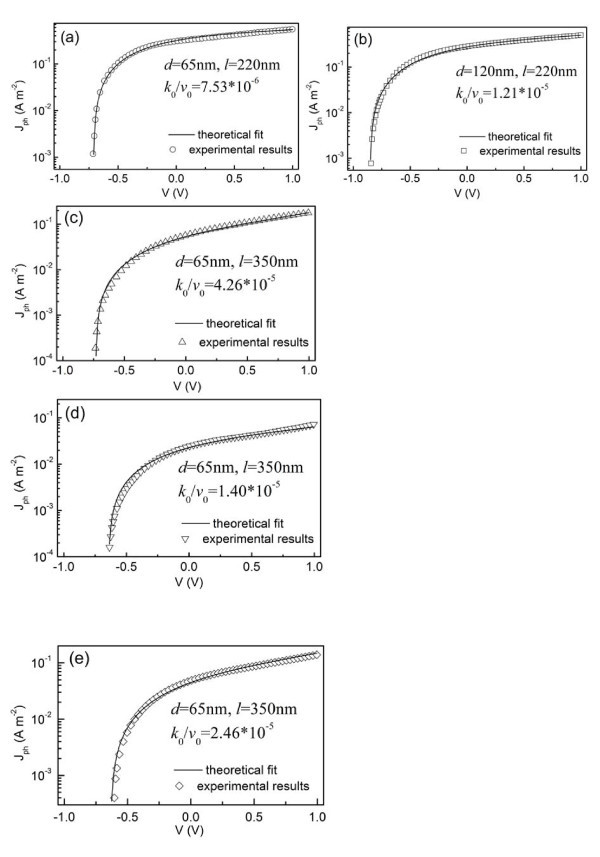
**The measured and fitted photocurrent-voltage curves for ITO/TiO_2_/MEH-PPV/Au devices**. (a-c) Panels are for the devices with different TiO_2 _and MEH-PPV layer thicknesses measured under the same illumination intensity; while (c, d) panels are used to show the influence of illumination intensity on the same device. The incident intensity was 15.85 mW/cm^2 ^(a-c), 3.0 mW/cm^2 ^(d) and 9.6 mW/cm^2 ^(e). The *k*_0_/*v*_0 _values obtained by fitting the experimental data to Equation 15 are marked on the respective panels.

In order to calculate the electric fields *E*_p _and *E*_n_, the accumulated charge density at the D/A interface is assumed to be a constant and *Q=*1.0 × 10^-4 ^C/m^2 ^[33]. We find that *Q *has a weak influence on the calculated results by our model, for which the reason may be that the internal electric field in the devices is only slightly modified due to the band bending created by the accumulation of the charge carriers at the D/A interface [24]. Therefore, it is reasonable that we simply assume *Q *is a constant. In spite of the parameters *ε*_p _= 4*ε*_0 _which is comparable to *ε*_p _= 3*ε*_0 _[45], *ε*_n _= 55*ε*_0 _[49], *α*_p_(λ = 470 nm) = 10^5 ^cm^-1^, and *L*_p _= 15 nm [12,13,50], there are still three parameters (i.e., *λ*, *k*_0_/*v*_0_, and *β*) needed to obtain *J*_ph _by Equation 15. Our calculated data revealed that the shape of *J*_ph_-*V *curve is strongly dependent on the values of *λ*, but less dependent on the values of *k*_0_/*v*_0 _and *β*. Therefore, the parameter *λ *can be first obtained by curve fitting taking the order of magnitude of 10^-5 ^for *k*_0_/*v*_0 _and that of 10^-3 ^for *β*; then, the values of *k*_0_/*v*_0 _and *β *can be obtained by the best fit. In our model, we take *λ *= 3 and *β *is a constant with a value of 5 × 10^-3 ^V^-2^. Finally, the ratio *k*_0_/*v*_0 _is the only adjustable fit parameter in fitting the experimental photocurrent. Since *k*_0 _and *v*_0 _are zero-field recombination rate constants, the ratio *k*_0_/*v*_0 _is independent of the electric field. However, the ratio *k*_0_/*v*_0 _depends on the used materials or the geometry of the devices [48] such as the TiO_2 _(polymer) film thickness as shown in Figure 4.

**Figure 5:**
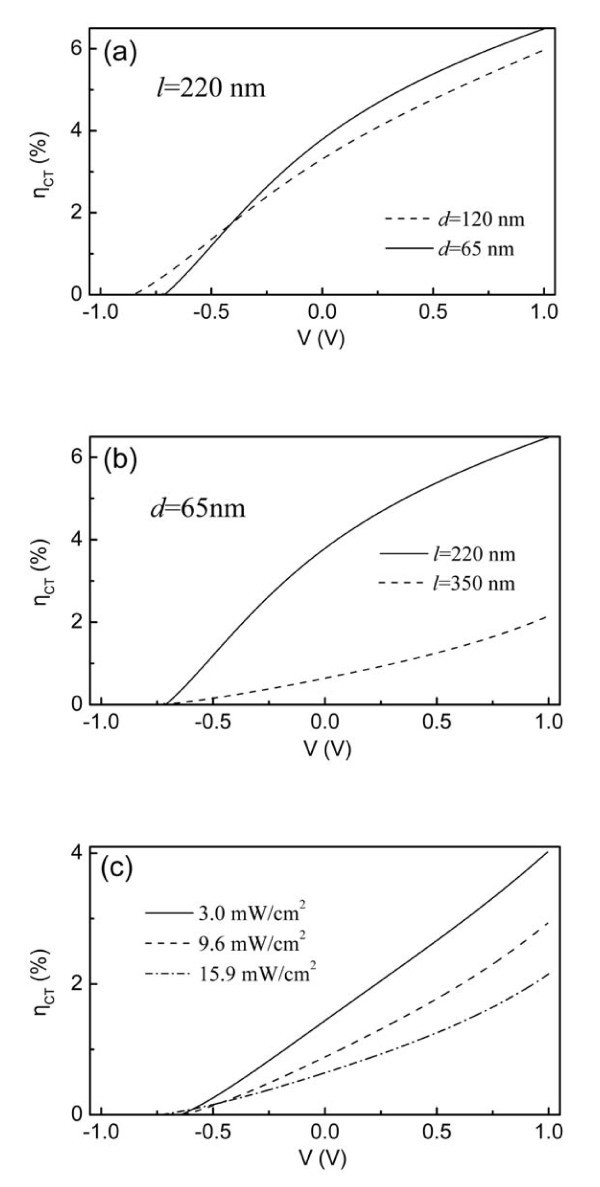
**The calculated *η*_CT _as a function of applied voltage *V *with deferent *l *(a), *d *(b) and various illumination intensities (c)**.

Note that, all the following theoretical curves were obtained by considering the experimentally determined compensation voltage *V*_0_. As shown by the solid lines in Figure 4, the excellent fits to the photocurrent-voltage characteristics of three types devices are obtained using the parameters described above. During the calculations, we use different *k*_0_/*v*_0 _values to fit the photocurrent-voltage characteristics of the differently structured devices (Figure 4a,b,c) and the same cell under the varied illumination intensities (Figure 4c,d,e). In Figure 4, it can be seen that the photocurrent increases as the applied voltage turns from reverse to forward direction, and subsequently tends to saturate at higher forward voltages. This phenomenon can be attributed to the dependence of the exciton dissociation efficiency *η*_CT _on the internal electric field (Equation 14), since the efficiency *η*_ED _of exciton dissociation by charge transfer at the D/A interface is constant and the efficiency *η*_CC _of charge collection at electrodes is equal to 1 (Equation 4) [39]. As suggested from Figure 3a, the exciton dissociation efficiency at the D/A interface increases with increasing the forward electric field strength (i.e., the forward applied voltage), and finally approach unit when the forward electric field strength is large enough. In order to examine the dependence of *η*_CT _on the applied voltage *V*, the TiO_2 _and polymer film thicknesses and illumination intensity, we plot the expression *η*_CT _from Equation 14 for all devices, as shown in Figure 5.

Figure 5a shows that, for the devices with different TiO_2 _thicknesses (*d*), when *V *- *V*_0 _> 0, i.e., *E*_p_(*E*_n_) > 0, *η*_CT _increases with the increasing forward applied voltage, indicating that the forward electric field is beneficial to the exciton dissociation efficiency as indicated in Figure 3a. When the forward electric field is large enough (*V *> -0.4 V here), *η*_CT _for the device with *d *= 65 nm is larger than the calculated one for the device with *d *= 120 nm, which is in agreement with the result that a thicker TiO_2 _film leads to a higher series resistance and a lower photocurrent [11].

As for the devices with different polymer thicknesses (*l*) (Figure 5b), the similar dependence of the dissociation efficiency *η*_CT _on the applied voltage is obtained, i.e., a higher the forward electric field results in a larger exciton dissociation efficiency *η*_CT_. However, the thicker polymer film leads to a much smaller exciton dissociation efficiency in the whole applied voltage region. It is very likely due to the slower hole transfer rate in the polymer film as a result of the weakened internal electric field by the increased polymer film thickness, which leads to the smaller exciton dissociation rate at the D/A interface and further the lower exciton dissociation efficiency [5,51].

Figure 5c shows the influences of various incident intensities on the exciton dissociation efficiency *η*_CT_. It is found that *η*_CT _decreases with increasing the incident intensity at same applied voltage. The similar phenomenon that the efficiency of charge separation per incident photon decreases with increasing the incident light intensity has also been observed in bilayer TiO_2_/PdTPPC [16] and TiO_2_/P3HT [40] cells in the absence of internal electric field, and was attributed to the occurrence of exciton-exciton annihilation within the polymer layer. In our case, this phenomenon can be understood as follows. Although a higher incident intensity creates more excitons in the polymer layer and generates higher free electron and hole densities at the D/A interface, the higher densities of the charge carriers at the interface increases the charge recombination probability at the same time; moreover, as discussed above, the increasing forward applied voltage will enhance the exciton dissociation efficiency at the D/A interface. In other words, there is a competition between exciton dissociation and charge recombination at the D/A interface and the last result is that the exciton dissociation efficiency *η*_CT _decreases as shown in Figure 5. This important result indicates that the increase in the photocurrent density under a higher incident light intensity is due to the increase in exciton density rather than the increase in the exciton dissociation efficiency, which is useful to optimize device performance.

## Conclusions

An analytical model for the photocurrent-voltage (*J*_ph_-*V*) characteristics of the bilayer polymer/TiO_2 _photovoltaic cells is developed, where the generation of free charges takes place via dissociation of photogenerated excitons. The model describes the dependence of photocurrent generation on the device geometry and gives an analytical expression for the exciton dissociation efficiency. The experimental *J*_ph_-*V *data of the MEH-PPV/TiO_2 _devices are satisfactorily fitted to the model. Results show that increasing TiO_2 _or the polymer layer in thickness will reduce the exciton dissociation efficiency *η*_CT _in the device and further the photocurrent. It is found that the photocurrent is determined by the competition between the exciton dissociation and charge recombination at the D/A interface, and the increase in photocurrent under a higher incident light intensity is due to the increased exciton density rather than the increase in the efficiency *η*_CT_. Our results indicate that a thinner polymer layer combined with a thinner TiO_2 _layer favors the higher exciton dissociation efficiency in the bilayer devices. The model will provide information on optimization of device performance by investigating the effects of material parameters on device characteristics.

## Abbreviations

A: acceptor; CIMPS: controlled intensity modulated photo spectroscopy; D: donor; HOMO: highest-occupied molecular orbital; ITO: indium tin oxide; LED: light-emitting diode; MC: Monte Carlo; PV: photovoltaic; TiO_2_: titanium dioxide.

## Competing interests

The authors declare that they have no competing interests.

## Authors' contributions

CC performed the experiments, developed the theory model, and drafted the manuscript. FW participated the theoretical analysis. HG and WS participated the device preparation. MW conceived of the study, and participated in its design and coordination. All authors read and approved the final manuscript.
